# Tumor RNA-loaded nanoliposomes increases the anti-tumor immune response in colorectal cancer

**DOI:** 10.1080/10717544.2021.1954727

**Published:** 2021-07-21

**Authors:** Dandong Dai, You Yin, Yuanbo Hu, Ying Lu, Hongbo Zou, GuangZhao Lu, Qianqian Wang, Jie Lian, Jie Gao, Xian Shen

**Affiliations:** a Department of Gastrointestinal Surgery, The Second Affiliated Hospital and Yuying Children’s Hospital of Wenzhou Medical University, Zhejiang, China; b Department of Pharmaceutical Sciences, Naval Medical University, Shanghai, China; c Institute of Translational Medicine, Shanghai University, Shanghai, China; d Department of Neurology, Changzheng Hospital of Naval Medical University, Shanghai, China; e Department of Gastrointestinal Surgery, The First Affiliated Hospital of Wenzhou Medical University, Zhejiang, China; f Department of Medical Oncology, Hangzhou First People’s Hospital, Zhejiang, China; g Department of Pathology, Shaoxing Shangyu People’s Hospital, Zhejiang, China

**Keywords:** Colorectal cancer, nanoliposome, vaccine, chemotherapy, RNA vaccine, LPD

## Abstract

**Purpose:**

Tumor RNA vaccines can activate dendritic cells to generate systemic anti-tumor immune response. However, due to easily degraded of RNA, direct RNA vaccine is less effective. In this study, we optimized the method for preparing PEGylated liposom-polycationic DNA complex (LPD) nanoliposomes, increased encapsulate amount of total RNA derived from CT-26 colorectal cancer cells. Tumor RNA LPD nanoliposomes vaccines improved anti-tumor immune response ability of tumor RNA and can effectively promote anti-tumor therapeutic effect of oxaliplatin.

**Methods:**

Total tumor-derived RNA was extracted from colorectal cancer cells (CT-26 cells), and loaded to our optimized the LPD complex, resulting in the LPD nanoliposomes. We evaluated the characteristics (size, zeta potential, and stability), cytotoxicity, transfection ability, and tumor-growth inhibitory efficacy of LPD nanoliposomes.

**Results:**

The improved LPD nanoliposomes exhibited a spherical shape, RNA loading efficiency of 9.07%, the average size of 120.37 ± 2.949 nm and zeta potential was 3.34 ± 0.056 mV. Also, the improved LPD nanoliposomes showed high stability at 4 °C, with a low toxicity and high cell transfection efficacy toward CT-26 colorectal cancer cells. Notably, the improved LPD nanoliposomes showed tumor growth inhibition by activating anti-tumor immune response in CT-26 colorectal cancer bearing mice, with mini side effects toward the normal organs of mice. Furthermore, the effect of the improved LPD nanoliposomes in combination with oxaliplatin can be better than that of oxaliplatin alone.

**Conclusion:**

The improved LPD nanoliposomes may serve as an effective vaccine to induce antitumor immunity, presenting a new treatment option for colorectal cancer.

## Introduction

Colorectal cancer is the third most common cancer and the second most common cause of cancer-related death worldwide (Sung et al., 2021). According to global statistics for 2020, there were 19.3 million new cancer cases; colorectal cancer accounted for 10.0% of these cases. Deaths due to colorectal cancer comprise 9.4% of nearly 10 million cancer-related deaths (Sung et al., 2021). At present, the treatment of colorectal cancer primarily involves surgical resection-based treatment; other treatment approaches include chemotherapy, radiotherapy, and targeted therapy (Benson et al., 2021).

Cancer vaccines are a promising treatment for cancer therapy which is designed to kill or control cancer cells by activating the immune system with cancer antigens (Sayour et al., 2018). One category of cancer vaccine, dendritic cells (DCs) vaccines, has received extensive attention due to its potent activity by activation of immune systems through stimulation of DCs with peptide, DNA or RNA (Rossi et al., 2021). Among all DCs vaccines, RNA DC vaccines, produced by RNA pulsed DCs vaccines, are capable of bypassing MHC classification restriction, eliciting immunogenicity without the need for adjuvant, exhibiting a high safety profile without integration into the genome (Miao et al., 2021). Furthermore, tumor vaccines loaded with a single tumor antigen may induce immunosuppression and immune evasion, while identifying tumor specific antigens can require expensive and laborious procedures. Therefore, the use of whole tumor cell antigens is currently considered to be promising, potentially effective methods (Heiser et al., 2001a, 2001b; Naka et al., 2008; Miao et al., 2021). Several RNA DC vaccines through the stimulation of DCs with tumor RNA extracted from cancer cells have been shown to be effective in the treatment of colorectal cancer (Heiser et al., 2001a, 2001b; Naka et al., 2008). However, these RNA DC vaccines need to be produced by a complex procedure consisting of isolation of DCs and stimulation of DCs with RNA, and thus it is necessary to develop a much simpler and more effective alternative approach.

Nanocarriers have been developed as efficient delivery vehicles for RNA, which can efficiently deliver tumor antigen RNA directly to DCs and induce immune responses (Markov et al., 2017). Compared with common RNA DC vaccines, nanocarriers delivering tumor antigen RNA possess several advantages: (1) Avoiding the complex procedure of isolation of DCs; (2) Nanocarriers can effectively increase the stability of RNA; (3) Nanocarriers are more readily recognized and engulfed by DCs compared with free drugs. Cationic liposomes are one of the most studied carriers in gene therapy (Ma et al., 2021). We have previously successfully developed PEGylated liposome-polycation DNA complex (LPD) for siRNA and RNA delivery (Gao et al., 2011, 2012, 2013). Therefore, in this study, we extracted the total RNA of colorectal cancer cells and loaded them on the improved LPD nanoliposomes to construct an RNA LPD nanoliposomes vaccine. In this study, we evaluated the characteristics (size, zeta potential, and stability), cytotoxicity, transfection ability, and tumor-growth inhibitory efficacy of the LPD nanoliposomes. This study provides a new tumor vaccine with high-efficiency and low toxicity toward the immunotherapy of colorectal cancer.

### Materials, cell culture, and mice

(N-[1-(2,3-Dioleoyloxy)propyl]-N,N,N-trimethylammonium methyl-sulfate) (DOTAP) and 1,2-distearoyl-sn-glycero-3-phosphoethanolamine-N-carboxy(polyethylene glycol)-2000 (sodium salt) (DSPE-PEG(2000) Carboxylic Acid) were purchased from Avanti Inc. (USA; purity ≥98% for both); cholesterol, Shanghai McLean Biochemical Technology Co. Ltd. (Shanghai, China; purity ≥98%); DEPC-treated water, RPMI-1640 medium, sodium pyruvate solution, 1 M HEPES solution, 200 mM ʟ-Glutamine solution (100X), purinamycin, and lipopolysaccharide (LPS), Dalian Meilun Biotechnology Co. Ltd. (Dalian, China); chloroform, Sinopharm Shanghai Chemical Reagent Co. Ltd. (Shanghai, China); protamine sulfate salt from salmon and calf thymus DNA, Sigma (USA); MEM non-essential amino acid solution (100X) and fetal bovine serum (FBS), Gibco (USA); TRIzol™ reagent, PBS, Dynabeads™ Untouched™ Mouse CD8 Cell Kit, and Lipofectamine™ 3000 transfection reagent, CD8 Polyclonal Antibody, Thermo Fisher Scientific Inc. (USA); agarose, Biofroxx (Germany); TAE (50X) solution, Ranjeck Technology Co. Ltd. (Hefei, China); DL 2000 DNA Marker, TAKARA Biomedical Technology (Beijing) Co. Ltd. (Beijing, China); 10,000 X Solarred Nucleic Acid Dye, Beijing Solarred Technology Co. Ltd. (Beijing, China); cell culture dishes, Corning Company (USA); LV-Enhance (virus infection enhancement solution), Shanghai Fu Baiao Biological Technology Co. Ltd (Shanghai, China); lentivirus (ZSgreen), General Biosystems (Anhui) Co. Ltd. (Hefei, China); CCK-8, Dongren Chemical Technology (Shanghai) Co. Ltd. (Shanghai, China); red blood cell lysate, Beijing SolarBio Technology Co. Ltd. (Beijing, China); restructuring IL-4 (rm IL-4) in mice, recombinant mouse IL-2 (RM IL-2), recombination mouse GM-CSF (RMGM-CSF), and recombination mouse GM-CSF (RMGM-CSF), PeproTech Inc. (USA); PE-CY7-CD11c mAb, PE – MHC II mAb, APC – CD80 mAb, PE-CD86 mAb, FITC-CD11C mAb, APC-CD40 mAb, and PE-CD8 mAb, Ebioscience Inc. (USA); Cyto Tox96^®^ Non-Radioactive Cytotoxicity Assay Kit, Promega Inc. (USA); mouse lymphocyte isolation solution, Tianjin Haoyang Biological Products Co. Ltd. (Tianjin, China); mouse interferon γ (IFN-γ) ELISA kit, alanine aminotransferase (ALT) colorimetric test box, aspartate aminotransferase (AST) colorimetric test box, creatinine (CR) colorimetric test box (sarsine oxidase method), and urea colorimetric test box (urease method), Wuhan Elarite Biotechnology Co. Ltd. (Wuhan, China). An extruder set with a holder/heating block was purchased from Avanti Inc.

CT 26 (H-2d), an undifferentiated low-immunogenic murine colorectal adenocarcinoma cell line, was purchased from Shanghai Xuji Biotechnology Co. Ltd. (Shanghai, China). The cells were cultured in RPMI-1640 medium supplemented with 10% fetal bovine serum (FBS), 110.0 mg/L sodium pyruvate solution, 2383.0 mg/L HEPES solution, (1X) MEM non-essential amino acid solution, and 300.0 mg/L ʟ-glutamine solution.

All animal procedures were performed in accordance with the guidelines of the Committee on Animals of the Naval Medical University (Shanghai, China). All Balb/c (male, 18–20 g, 4–5 weeks of age) were purchased from Shanghai Jihui Experimental Animal Feeding Co. Ltd. (Shanghai, China). We tried our best to relieve the suffering of experimental mice in this study. Before use in the experiments, the mice were allowed to acclimate for 7 days.

### Isolation of RNA

RNA was isolated by a standard TRIzol-based method. Briefly, total tumor-derived RNA from CT-26 cells was isolated using the TRIzol reagent, following the manufacturer’s instructions. RNA was spectrophotometrically quantified at 260 and 280 nm using a NanoDrop 2000 spectrophotometer.

### Preparation and characterization of LPD nanoliposomes

LPD nanoliposomes were prepared and slightly modified according to previously reported methods (Powell et al., 2017). Briefly, DOTAP/cholesterol liposome (1:1 M ratio, 4.5 mM) was prepared by the thin film hydration method, followed by membrane extrusion with a hand-held extruder (Avestin, Ottawa). The extrusion was performed in a stepwise manner using progressively decreasing pore-sized membranes (from 200, 100, and 50 nm) (Nucleopore, Whatman), with 10–20 cycles per pore-size. Briefly, 63 μL DOTAP/cholesterol liposome, 12 μL protamine (2 ug/μL), and 10 μL DEPC-treated water were mixed and incubated for 10 mins at 25 °C to form complex A. 45 μL RNA (0.5 μg/μL), 1.2 μL calf thymus DNA (10 ug/μL), and 30 μL DEPC-treated water were mixed and incubated for 10 mins at 25 °C to form complex B. Then, 3.2 μL (10 ug/μL) of DSPE-PEG-2000 carboxylic acid was added to the complex B to form complex C, the complex A and C at 50 °C for 1.5 mins. Then, complex A and complex C were mixed and incubated for 10 min at 25 °C to produce complex D. Finally, the complex D was placed at 55 °C for 10 min and cooled at 25 °C for 10 min to produce the final LPD nanoliposomes. The size and zeta potential of nanoliposomes were measured using the Zetasizer Nano ZS (Malvern Instruments Ltd., Worcestershire, UK). After the nanoliposomes were dropped onto a copper grid coated with a carbon membrane, stained by 2% phosphotungstic acid (PTA) and dried, the size and morphology of nanoliposomes were determined using transmission electron microscopy (TEM; JEM2100F, JEOL, Japan).

Noticeably, in our research, we have improved the traditional LPD preparation approach mainly in the following two ways: (1) Prior to mixing A (DOTAP/Chol cationic liposome and protamine) and B (calf thymus DNA and RNA), DSPE-PEG (2000) Carboxylic Acid was added to B to form C. (2) Before A and C were mixed, they were heated at 50 °C for 1.5 minutes.

### Gel electrophoresis of LPD nanoliposomes

The RNA binding ability of LPD nanoliposomes was evaluated by gel retardation assay. The LPD nanoliposomes were loaded into individual wells of 1% agarose gel, electrophoresis was carried out at 120 V for 30 min. The resulting RNA migration pattern was revealed under UV irradiation.

### The stability assay of LPD nanoliposomes

The LPD nanoliposomes prepared were stored at 4 °C, and gel electrophoresis was used to assess the RNA integrity of LPD nanoliposomes at different time points. Furthermore, the LPD nanoliposomes were also mixed with FBS at a volume ratio of 1:1 and stored at 37 °C, and gel electrophoresis was used to assess the RNA integrity of LPD nanoliposomes at different time points.

### Construction of CT-26 cells expressing green fluorescent protein

We constructed CT-26 cells expressing green fluorescent protein as described below. Briefly, the constructed FV115 ZSGreen lentivirus (General Biosystems Co. Ltd. Hefei, China) was added to the 12-well culture plate, and then collected by high-speed centrifugation. After centrifugation, the supernatant was discarded. CT-26 cells suspended in RPMI-1640 complete medium containing 10% FBS were added and centrifugated. After centrifugation, 3 μg/mL puromycin was added for drug screening. After screening, the green fluorescence expression of CT-26 cells was observed under a fluorescence microscope (IX73 fluorescence microscope OLYMPUS, Japan).

### Cytotoxicity assays of LPD nanoliposomes

The cytotoxic effect of LPD nanoliposomes on CT-26 cells and DCs was detected using the CCK-8 kit. Briefly, cells were plated on the 96-well plate at a density of 0.4 × 10^5^/mL for CT-26 cells and 2 × 10^5^/mL for DCs for 24 h or 48 h. After then, different concentrations of LPD nanoliposomes were added to the cells for 24. Finally, 10 μL of CCK-8 solution was added to each well. After incubation for approximately 4 h, the absorbance of each well was read at 450 nm using a microplate reader (Thermo Scientific, USA).

### Transfection of LPD nanoliposomes

CT-26 cells at a density of 0.5 × 10^5^/mL were plated in the laser confocal culture dish for 24 h in an incubator at 37 °C. Different concentrations of LPD nanoliposomes (1 mL LPD solution contained 0.96 mg DOTAP/cholesterol liposome, 0.15 mg protamine, 0.14 mg RNA, 0.07 mg calf thymus DNA and 0.19 mg DSPE-PEG (2000) Carboxylic Acid. The content of LPD and RNA in the solution were 1.5 and 0.14 mg, respectively. Moreover, the RNA content in each group was set as 1, 2, 5, 8, 10, 13, 19, and 24 μg/mL for cytotoxicity test, and LPD content in appropriate group was 11, 21, 54, 86, 107, 139, 203, and 257 μg/mL.) were added to each well and incubated for 24 or 48 h. RNA transfection using Lipofectamine^TM^ 3000 was performed according to the manufacture’s standard protocols. The transfected RNA used this assay was extracted from CT-26 cells expressing green fluorescent protein established as above. The green fluorescence expression of CT-26 cells was observed under a fluorescence microscope (IX73 fluorescence microscope OLYMPUS, Japan) and Leica TCS SP2 confocal microscope (Leica, Germany).

### Preparation of bone marrow-derived DCs and spleen-derived CD8+ T cells

Primary bone marrow DCs were extracted from mouse bone marrow precursors based on previously reported methods (Heiser et al., 2001a; Li & Huang, 2006). In brief, tibias and femurs from BALB/c mice (4-to 5-week-old, male) were flushed to gain bone marrow and then erythrocytes were depleted using commercial lysis buffer (Solarbio, Beijing, China). Cells were washed twice using serum-free RPMI-1640 medium and cultured with RPMI-1640 medium supplemented with 10% FBS, 10 ng/mL recombinant murine GM-CSF, and 10 ng/mL recombinant murine IL-4 in six-well plates (1 × 10^6^ cells/mL; 4 mL per well) at 37 °C under 5% CO_2_. Half of the medium was replaced with fresh cytokines containing rmGM-CSF and rmIL-4 without discarding any cells on days 3 and 5. On day 7, LPS (1 µg/mL) was added to immature DCs (iDCs) and incubated for 24 h to obtain mature DCs (mDCs).

Primary CD8+ T cells were extracted from the mouse spleens according to previously reported methods. Briefly, spleens from male BALB/c mice were aseptically removed and erythrocytes were depleted with a commercial lytic buffer. Mouse lymphocytes were isolated with a commercial mouse lymphocyte isolate, and CD8+ T cells were isolated using a CD8 immunomagnetic bead kit. The cells were cultured in RPMI-1640 medium buffer containing 10% FBS, 110.0 mg/L sodium pyruvate solution, 2383.0 mg/L HEPES solution, (1X) MEM non-essential amino acid solution, 300.0 mg/L ʟ-glutamine solution, and 100 ng/mL recombinant murine IL-2.

### Flow cytomety

Flow cytometry was used to detect the effect of LPD nanoparticles on the protein expression of DCs. Briefly, DCs were incubated with 8 μg/mL LPD nanoliposomes for 24 h. After that, DCs were digested with 0.05% EDTA in PBS for 6 min, collected, placed in a centrifuge tube, and centrifuged at 1700 rpm for 10 min. The supernatant was discarded. Next, 4 μg of fluorescent antibodies (CD40, CD86, MHC II, CD80, and CD11C) were added for surface expression and incubated at 4 °C for 40 min in the darkness. The cells were washed twice with PBS containing 2% FBS and centrifuged at 1700 rpm for 10 min. Finally, 500 μL of PBS solution was added to resuspend the cells for flow cytometry (BD Canto10c, USA).

CD8+ T cell maturity was determined using flow cytometry (BD Canto10c, USA). Briefly, CD8+ T cells were collected and placed in a centrifuge tube and centrifuged at 1700 rpm for 10 min; the supernatant was discarded. Next, 4 μg of fluorescent CD8 antibody was added for surface expression and incubated at 4 °C for 40 min in the darkness. The cells were washed twice with PBS containing 2% FBS, centrifuged at 1700 rpm for 10 min, resuspended with 500 μL of PBS solution, and analyzed by flow cytometry.

### Cytotoxic T-Lymphocyte (CTL) killing assay

T cell cytotoxicity was evaluated using the Cyto Tox 96 Non-Radioactive Cytotoxicity Assay Kit according to the manufacturer’s instructions. Briefly, iDCs treated with or without 8 μg/mL LPD nanoliposomes were induced by LPS to become mDCs. After that, mDCs were incubated with CD8+ T cells with a ratio of 1:20 (mDCs: CD8+ T) 7 days. T cells were then divided into two groups: (i) T cells stimulated by mDC alone (mDC-CTLs); (ii) T cells stimulated by mDC incubated with LPD nanoliposomes (LPD-mDC-CTLs). CT-26 cells at a density of 1 × 10^4^ cells/well were seeded in 96-well plates. The T cells were then cultured with CT-26 cells at various ratios (100:1, 50:1, 30:1, 20:1, 10:1) for 4 h. Finally, the absorbance was measured at 490 nm using a microplate reader (Thermo Scientific, USA). The following formula was used to calculate cell viability: (1 – [A – B]/[C – B]) × 100%, where A, B, and C are defined as the absorbance of experimental groups, total natural release groups, and largest release groups, respectively.

### The anti-tumor assay of LPD nanoliposomes

The therapeutic effect of LPD nanoliposomes on subcutaneous colorectal cancer in vivo was studied. Briefly, the CT-26 cancer cells were collected and resuspended in PBS at a concentration of 1 × 10^6^ cells/mL. For subcutaneous tumor implantation, the cells of 0.1 mL (1 × 10^5^ cells) were subcutaneously injected into the right lateral dorsal region of the CT-26 mice. One week later, the mice were intraperitoneally injected with various formulations (PBS, LPD nanoliposomes, RNA, at a dose of 27 μg RNA per mice) every 7 days for three times (7, 14 and 21 days). The tumor volume was calculated using the modified formula: 0.5 × (length × width^2^). Tumor volume and mouse weight were monitored every 2 days. The mice were euthanized when the tumor diameter reached 2.0 cm.

The therapeutic effect of LPD nanoliposome combined with oxaliplatin on subcutaneous colorectal cancer in vivo was studied. Briefly, the CT-26 cancer cells were collected and resuspended in PBS at an appropriate concentration of 1 × 10^6^ cells/mL. For subcutaneous tumor implantation, the cells of 0.1 mL (1 × 10^5^ cells) were subcutaneously injected into the right lateral dorsal region of the CT-26 mice. One week later, the mice were intraperitoneally injected with various formulations (PBS, LPD nanoliposomes, LPD nanoliposomes combined with oxaliplatin, and oxaliplatin). LPD nanoliposomes were administrated at a dose of 27 μg RNA per mice for three times (at 7, 13, and 19 days). Oxaliplatin was administrated at a dose of 27 μg per mice five times (at 7, 10, 13, 16, and 19 days). The tumor volume was calculated as described above.

Before the euthanasia of the mice, the blood was obtained from the mice and submitted for comprehensive chemistry analysis by an automatic biochemical analyzer (OLYMPUS AU5421). The concentration of IFN-γ in the supernatant was detected using commercially available ELISA kits in accordance with the manufacturer’s instructions.

After the euthanasia of the mice, the tumor tissue, heart, liver, spleen, lung and kidney of the mice were excised and fixed with 4% paraformaldehyde. According to standard histological procedures, sections of the tumor tissues, heart, liver, spleen, lung, and kidney were stained with hematoxylin-eosin (H&E). The results were imitated using a Digital slice scanning system Precice 500 (Beijing Uniona Technology Co. Ltd., Beijing, China).

### Immunohistochemical staining

After the mice were euthanized, the spleens and tumors were taken, and the distribution of CD8+ T lymphocytes in the tissues was detected by immunohistochemistry. In short, 4% paraformaldehyde-fixed paraffin-embedded tissue blocks from tumor and spleen specimens were annotated by pathologists, cut into 5um sections, and mounted on positively charged glass slides. Use rabbit anti-mouse polyclonal monospecific antibody (CD8 polyclonal antibody), diluted 1:100, and stain each sample according to the standard protocol. All CD8 immunohistochemical slices are reviewed by the same senior pathologist. The review process is objective and independent. First, observe the full picture of the slices at low magnification (100×), and then select representatively at 200 times magnification Field of view, switch to a high-power lens (×400), randomly select 5 high-power fields, and count positive cells in each field. Quantification of positive CD8 + T cells was the average of the total of 5 positive cell fields using Image-Pro plus software (version 6.0). The expression of CD8 was scored and categorized as 0, +1, +2 or +3 according to the intensity of staining according to the reference and was analyzed by Fisher’s exact test.

### Statistical analysis

Data was analyzed by SPSS 13.0 (SPSS Inc, Chicago, IL, USA). For values that were normally distributed, a direct comparison between two groups was conducted by Student’s non-paired *t* test, two-tailed t-test, and one-way ANOVA with the Dunnett’s or Newman Keuls post-test was used to compare the means of three or more groups. *p* value of <.05 was considered statistically significant. *p<.05; **p<.01; ***p< .001; ****p<.0001; n.s. represents not significant (*p* > .05).

## Results

### Characterization of LPD nanoliposomes

**Figure 1.**
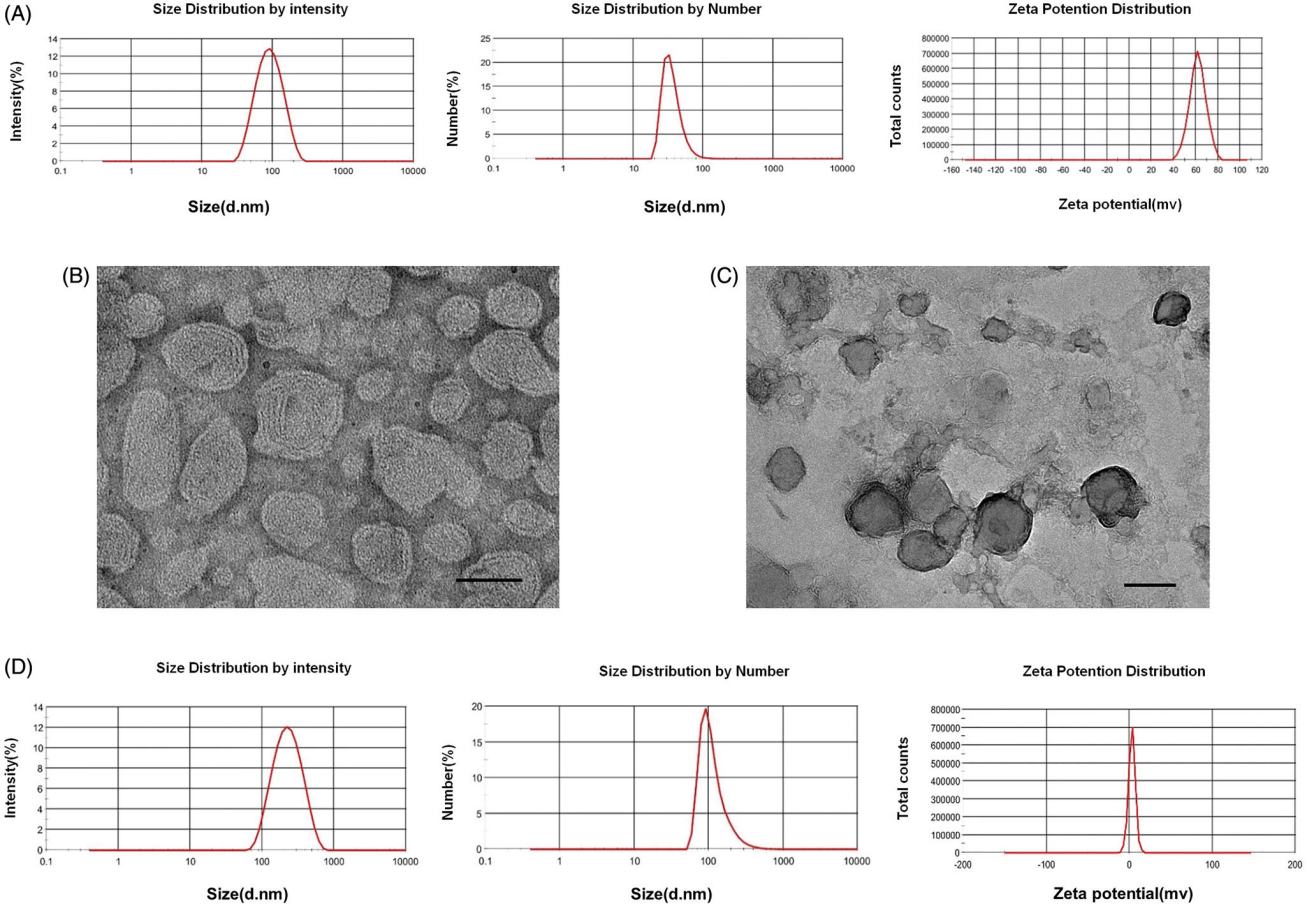
Characterization of DOTAP/chol nanoliposomes and LPD nanoliposomes. (A) Size distribution and zeta potential of DOTAP/chol nanoliposomes. One representative image is shown. (B) TEM images of DOTAP/chol nanoliposomes. Bars represent 50 nm. (C) TEM images of LPD nanoliposomes. Bars represent 100 nm. (D) Size distribution and zeta potential of LPD nanoliposomes. One representative image is shown.

Before the preparation of LPD nanoliposomes, DOTA/chol nanoliposomes should be prepared. As shown in Figure 1(A), DOTAP/Chol nanoliposomes possess the average size of 39.46 ± 4.889 nm, PDI of 0.249 ± 0.007, and zeta potential of 62.03 ± 1.974 mv. TEM images showed that DOTAP/Chol nanoliposomes were evenly distributed and spheric shape, with a particle size of approximately 50 nm (Figure 1(B)). Subsequently, LPD nanoliposomes were successfully prepared, with the average size of 120.37 ± 2.949 nm, PDI of 0.205 ± 0.002, and zeta potential of 3.34 ± 0.056 mV (Figure 1(D)). TEM images showed that the LPD nanoliposomes were evenly distributed, with spheric shape and the average size of approximately 100 nm (Figure 1(C)).

**Figure 2.**
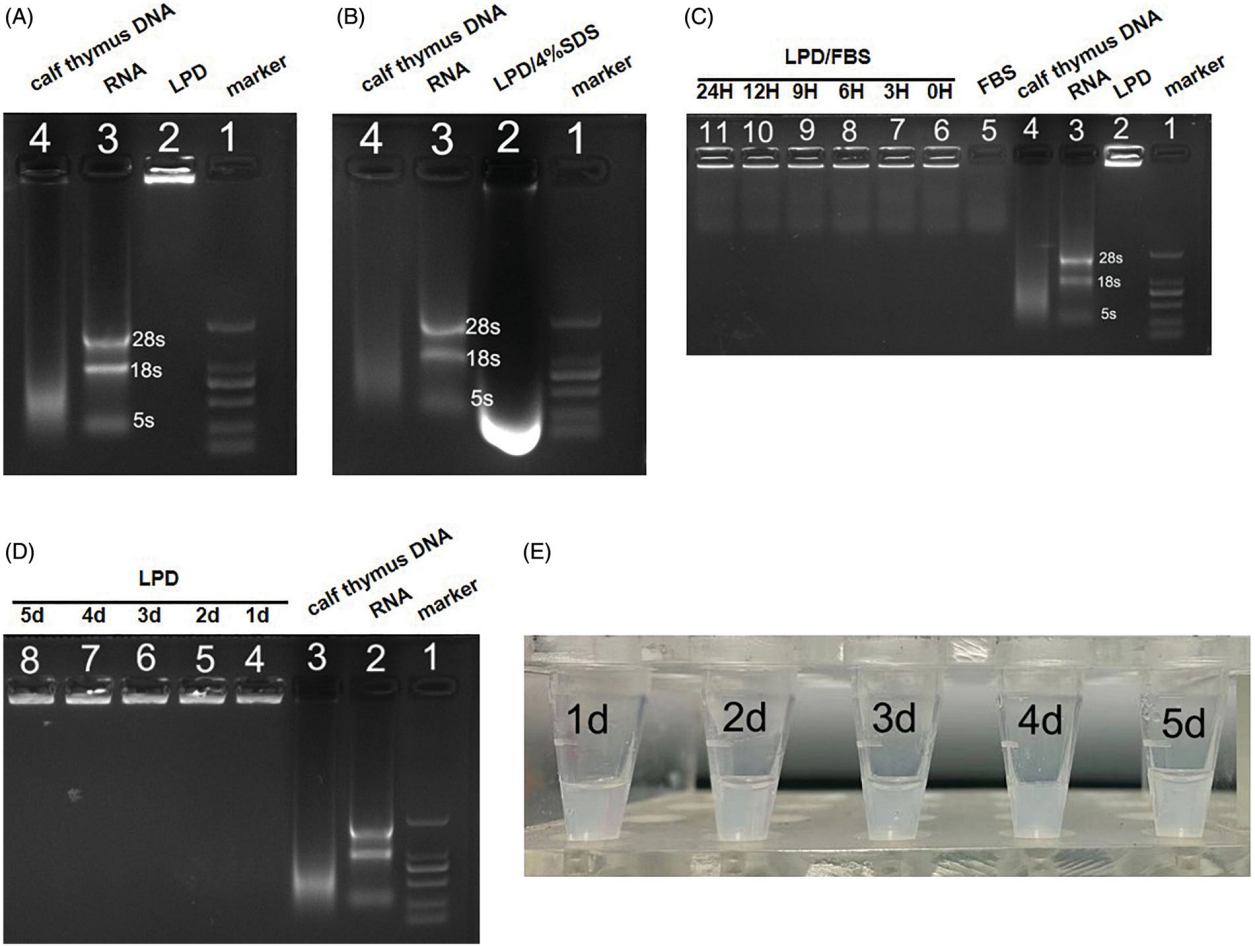
Gel retardation and stability assay of LPD nanoliposomes. (A) and (B) Gel retardation assay of LPD nanoliposomes. Lanes 1: DNA Marker; Lane 2: LPD nanoliposomes in the absence (A) or (B) presence of 4% SDS; Lane 3: pure RNA; Lane 4: calf thymus DNA. (C) Stability assay of LPD nanoliposomes. Lane 1: DNA Marker; Lane 2: LPD nanoliposomes; Lane 3: pure RNA; Lane 4: calf thymus DNA; Lane 5: FBS; Lane 6–11: LPD nanoliposomes mixed with FBS for 0, 3, 6, 9, 12, and 24 h. (D) Stability assay of LPD nanoliposomes. Lane 1: DNA Marker; Lane 2: pure RNA; Lane 3: calf thymus DNA; Lane 4–8: LPD nanoliposomes stored at 4 °C for 1, 2, 3, 4, and 5 days. (E) The images of LPD nanoliposomes stored at 4 °C for 1, 2, 3, 4, and 5 days.

Gel electrophoresis was used to evaluate the RNA encapsulation efficacy of LPD nanoliposomes. As shown in Figure 2(A), no RNA was found to escape from LPD nanoliposomes (see lane 2), indicating that the encapsulation efficiency of RNA is 100%. After being destroyed by 4% SDS, intact RNA was completely released (see lane 2, Figure 2(B)). LPD nanoliposomes were found to protect the RNA well and exhibit high stability in the presence of 50% serum during the incubation period of 0, 3, 6, 9, 12, and 24 h (see lane 6–11, Figure 2(C)). Also, LPD was found to be stable at 4 °C for 5 days, as indicated by the intact RNA encapsulation in the process of 1, 2, 3, 4 and 5 days and no aggregation in the solution (see lane 4–8, Figure 2(D,E)).

### Transfection efficiency of LPD nanoliposomes

**Figure 3.**
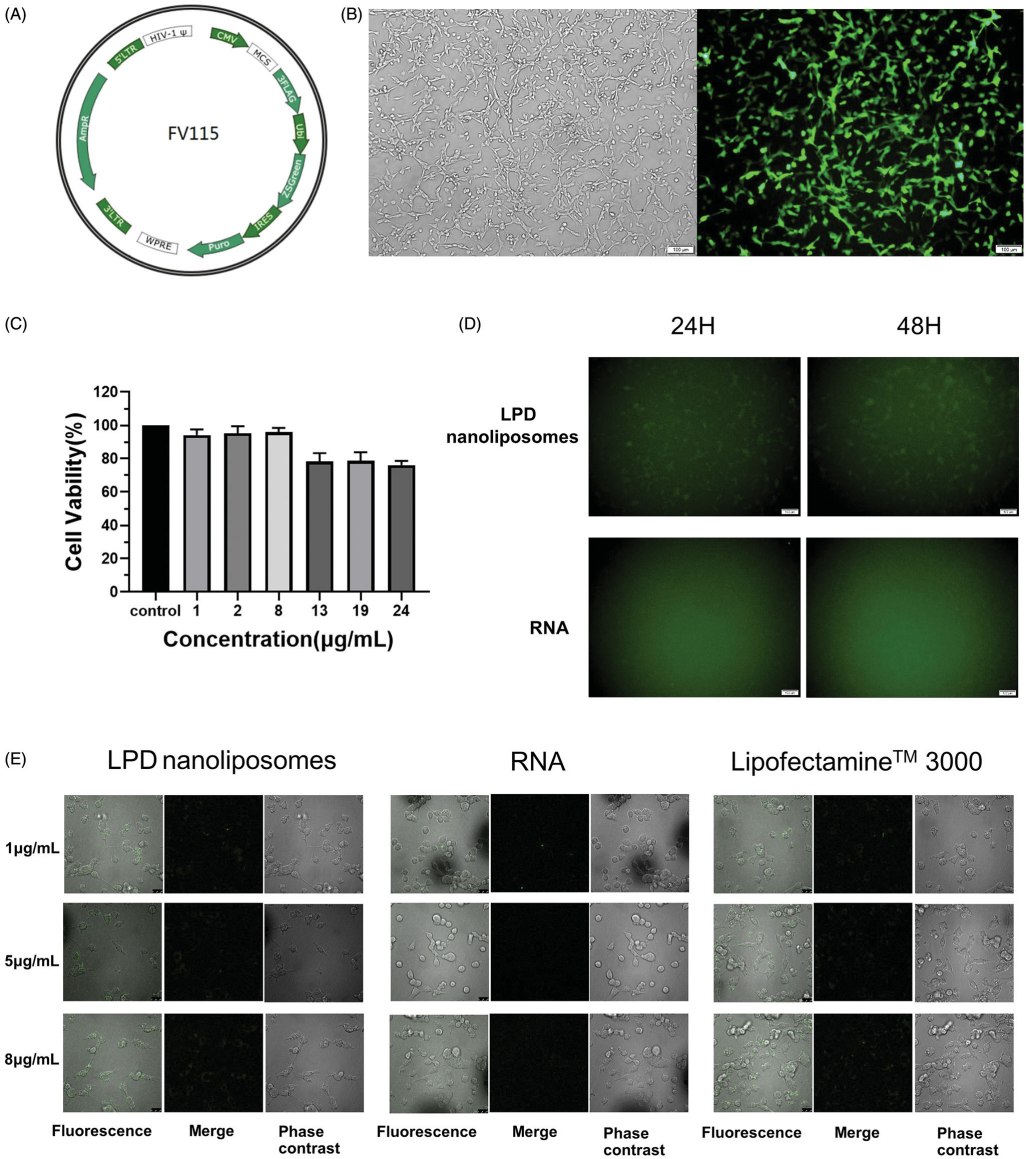
Transfection efficiency and cytotoxicity of LPD nanoliposomes. (A) The schematic diagram of FV115 lentiviral vector containing the green luciferase gene (ZSGreen). (B) The fluorescent images of CT-26 cells after transfection with FV115 lentivirus. (C) CCK-8 assays for determining the cytotoxicity of LPD nanoliposomes on CT-26 cells. The concentration of LPD was set as 1, 2, 8, 13, 19, and 24 μg/mL (RNA concentrations). Data are presented as mean ± SD (*n* = 5). (D) Transfection efficiency of LPD nanoliposomes and pure RNA at a concentration of 8 μg/mL under fluorescent microscope. (E) Transfection efficiency of LPD nanoliposomes and RNA under confocal microscope. The transfected RNA used this assay was extracted from CT-26 cells expressing green fluorescent protein.

Next, we evaluated the transfection efficiency of LPD nanoliposomes in CT-26 cells. Firstly, we constructed CT-26 cells stably transfected by lentivirus FV115 carrying the ZSGreen gene and expressing green fluorescent protein (Figure 3(A,B). After then, RNA was extracted from the stably transfected CT-26 cells and was loaded onto LPD nanoliposomes. As shown in Figure 3(C), LPD nanoliposomes did not affect the normal growth of the CT-26 cells, as reflected by the >75% cell viability in the presence of LPD nanoliposomes. As shown in Figure 3(D), LPD nanoliposomes can effectively transport RNA to CT-26 cells, and weak green fluorescence is observed, while green fluorescence is not observed in the pure RNA treatment group. Subsequently, we used confocal microscope to observe the transfection ability of LPD nanoliposomes (Figure 3(E)), and similar results were obtained, suggesting that our prepared LPD nanoliposomes possess potent transfection ability as the commercial transfection reagent Lipofectamine^TM^ 3000.

**Figure 4.**
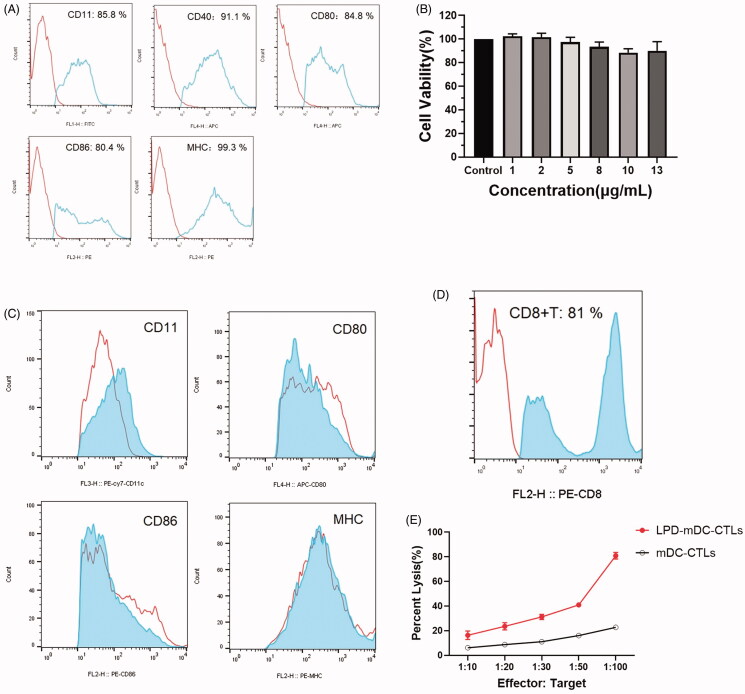
LPD nanoliposomes induce DCs and T cell activation *in vitro*. (A) Surface protein expression of mDCs measured by flow cytometry. (B) Proliferation assay of DCs in the presence of LPD nanoliposomes. (C) LPD nanoliposomes promote dendritic cell maturation *in vitro,* measured by flow cytometry. (D) Purity of positive CD8 T lymphocytes after isolation, as determined by flow cytometry. (E) Cytotoxicity assay for effector T cells (CTLs, cytotoxic T lymphocytes) activated by LPD nanoliposomes against CT-26 cells at different effect to target ratios. Data are presented as means ± standard deviations (*n* = 5).

### LPD nanoliposomes induce DCs and T cell activation

We collected primary DCs from the bone marrow of mice and verified the expression of CD11c, CD40, CD80, CD86 and MHC-II in the primary DCs (Figure 4(A)). As shown in Figure 4(B), we observed that LPD did not affect normal growth of DCs, and LPD stimulation increased the CD11 expression in DCs (Figure 4(C)). Because CD8+ T lymphocytes exhibit a strong ability to specifically inhibit tumor growth, we first evaluated the purity of CD8+ T cells from mice using magnetic activated cell sorting (MACS). The purity of CD8+ T lymphocytes was approximately 81.0% (Figure 4(D)). The cytotoxicity of LPD-mDC-CTLs, and mDC-CTLs targeting CT-26 cells was evaluated by Cyto Tox 96 Non-Radioactive Cytotoxicity Assay. The results showed that LPD-mDC-CTLs could be efficient at killing CT-26 cells (Figure 4(E)). These results suggested that LPD nanoliposomes could induce specific CTLs against CT-26 cells.

### *In vivo* antitumor effect of LPD nanoliposomes

**Figure 5.**
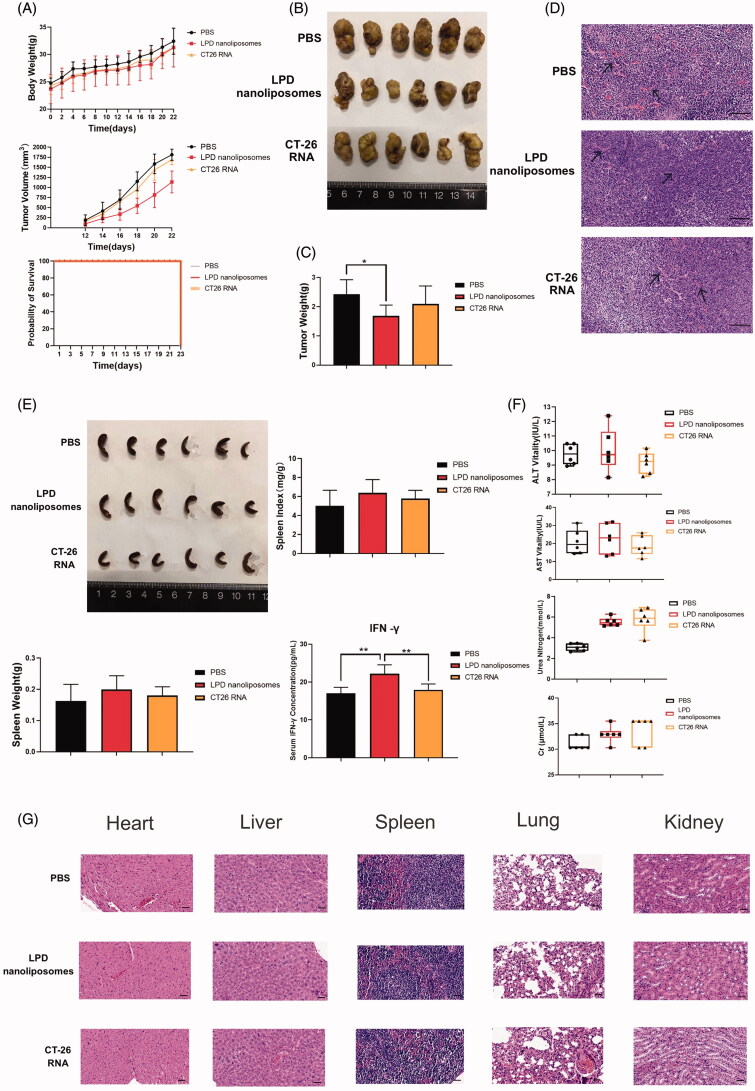
The therapeutic effect of LPD nanoliposomes on subcutaneous colorectal cancer *in vivo*. (A) The tumor growth curve, weight and survival curve of mice bearing subcutaneous colorectal cancer. For subcutaneous tumor implantation, the cells were subcutaneously injected into the right lateral dorsal region of the CT-26 mice. One week later, the mice were intraperitoneally injected with various formulations every 7 days for three times (7, 14, and 21 days). (B) The excised tumors from mice at the end point. (C) The weight of tumor excised from mice at the end point. (D) H&E staining of the excised tumors. Bars represent 200 μm. (E) The excised spleens, spleen weight, index, and serum IFN-γ concentrations of the mice at the end point were shown. (F) The biochemical index analysis of the mice at the end point. The two groups are compared with non-paired student’s *t* test. *p<.05; **p<.01. Data are presented as means ± SD (*n* = 6). (G) H&E staining of the heart, liver, spleen, lung, and kidney from mice at the end point. Bars represent 200 μm.

To determine the antitumor effects of LPD nanoliposomes, mice were immunized with LPD nanoliposomes or CT-26 RNA. Compared with the PBS and RNA treated groups, significantly delayed tumor growth was observed in LPD nanoliposomes-treated mice, and no significant difference was noted in the body weight and the survive during all the treatment process of the mice (Figure 5(A,B)). Weight analysis of the tumors showed that the weight of LPD nanoliposomes-treated tumors was lighter than that of PBS-treated tumors (*p*<.05), with a tumor inhibition rate of ∼30% (Figure 5(C)). It is well-known that the density of microvessels affects the prognosis of colorectal cancer, and pathological analysis after tumor HE is staining indicated that the density of tumor microvessels in the LPD nanoliposomes-treated group was significantly decreased (Figure 5(D)).

To explore whether LPD nanoliposomes could improve the immune response of mice, we measured the spleen index, spleen weight, and serum IFN-γ (Heiser et al., 2001a; Zhang et al., 2019). The results showed that the spleen weight and spleen index in the LPD nanoliposomes treated group was higher than the PBS or RNA treated groups (Figure 5(E)). Additionally, the IFN-γ concentration in the LPD nanoliposomes treated group was higher than the PBS or RNA treated groups (*p* < .01).

Furthermore, LPD nanoliposomes did not affect the normal liver and kidney function, as reflected by the ALT, AST, Urea and Cr concentrations (Figure 5(F)). In addition, according to the H&E staining of the heart, liver, spleen, lung, and kidney of the mice, no pathological changes were observed in the LPD nanoliposomes or RNA treated group (Figure 5(G)).

### *In vivo* anti-tumor effect of LPD nanoliposomes combined with oxaliplatin

**Figure 6.**
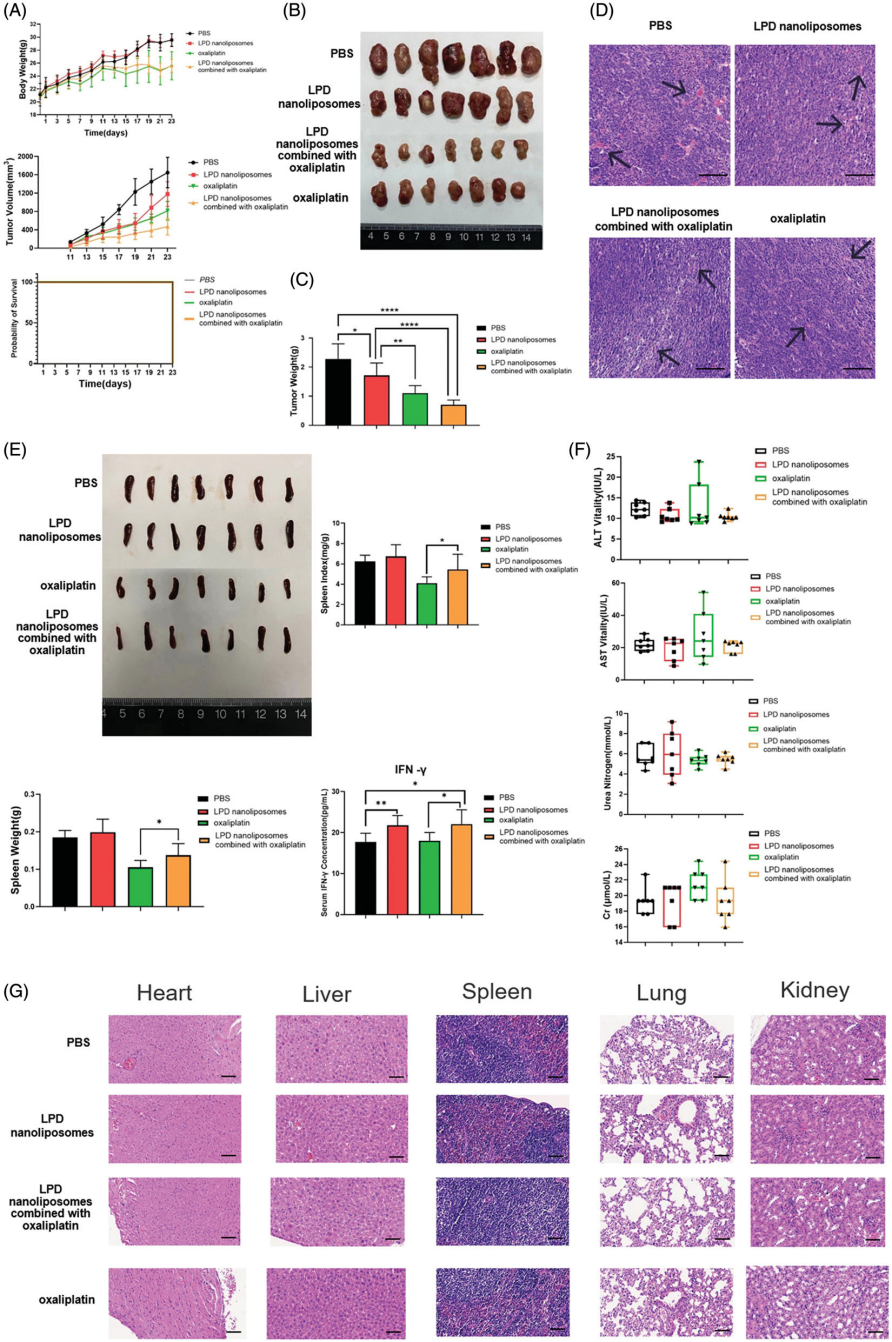
The therapeutic effect of LPD nanoliposomes combined with oxaliplatin on subcutaneous colorectal cancer *in vivo*. (A) The tumor growth curve, weight, and survival curve of mice bearing subcutaneous colorectal cancer. For subcutaneous tumor implantation, the cells were subcutaneously injected into the right lateral dorsal region of the CT-26 mice. One week later, the mice were intraperitoneally injected with various formulations (PBS, LPD nanoliposomes, LPD nanoliposomes combined with oxaliplatin, oxaliplatin). LPD nanoliposomes were administrated at a dose of 27 μg RNA per mice for three times (at 7, 13, and 19 days). Oxaliplatin was administrated at a dose of 27 μg per mice five times (at 7, 10, 13, 16 and 19 days). (B) The excised tumors from mice at the end point. (C) The weight of tumor excised from mice at the end point. (D) H&E staining of the excised tumors. Bars represent 200 μm. (E) The excised spleens, spleen weight, index and serum IFN-γ concentrations of the mice at the end point were shown. (F) The comprehensive chemistry analysis of the mice at the end point. One-way ANOVA with the Newman Keuls post-test was used to compare the means of three or more groups. *p<.05; **p<.01. Data are presented as means ± SD (*n* = 7). (G) H&E staining of the heart, liver, spleen, lung, and kidney from mice at the end point. Bars represent 200 μm.

The single RNA LPD has certain limitations in its anti-tumor effect through immunity in vivo, in order to further improve its anti-tumor effect, the anti-tumor effect of cancer vaccine could be significantly enhanced by combination with chemotherapy, and thus we aim to investigate the in vivo anti-tumor effect of LPD nanoliposomes combined with oxaliplatin. As shown in Figure 6(A,B), LPD nanoliposomes and oxaliplatin could significantly retard the progression of tumor growth, whereas LPD nanoliposomes combined with oxaliplatin showed the best anti-tumor effect. Furthermore, all the treatment did not affect the survival of mice. Consistently, weight analysis of the tumors showed that the weight of LPD nanoliposomes combined with oxaliplatin-treated mice was the lightest among all the groups (*p* < .0001), with a tumor inhibition rate of ∼69% (Figure 6(C)). Compared with the PBS control group, the tumor microvessel density of each treatment group was significantly reduced, and the tumor microvessel density of LPD nanoliposomes combined with oxaliplatin was reduced the most. (Figure 6(D)).

We also measured the spleen index, spleen weight, and serum IFN-γ concentration of the mice after various treatments (Figure 6(E)). The results showed that the spleen index and weight of LPD nanoliposomes combined with oxaliplatin-treated group were significantly increased compared with the oxaliplatin-treated group (*p* < .05). Compared with the PBS control group, the IFN-γ concentration of the LPD nanoliposomes group was higher (*p* < .05). Compared with the oxaliplatin group, the IFN-γ concentration of the combined treatment group was higher (*p* < .05). We evaluated the safety of all the formulations in mice (Figure 6(F,G)). In these studies, LPD nanoliposomes appeared to be safe based on organ function tests and end-organ histology.

### T cell infiltration in the spleen and tumor microenvironment

**Figure 7.**
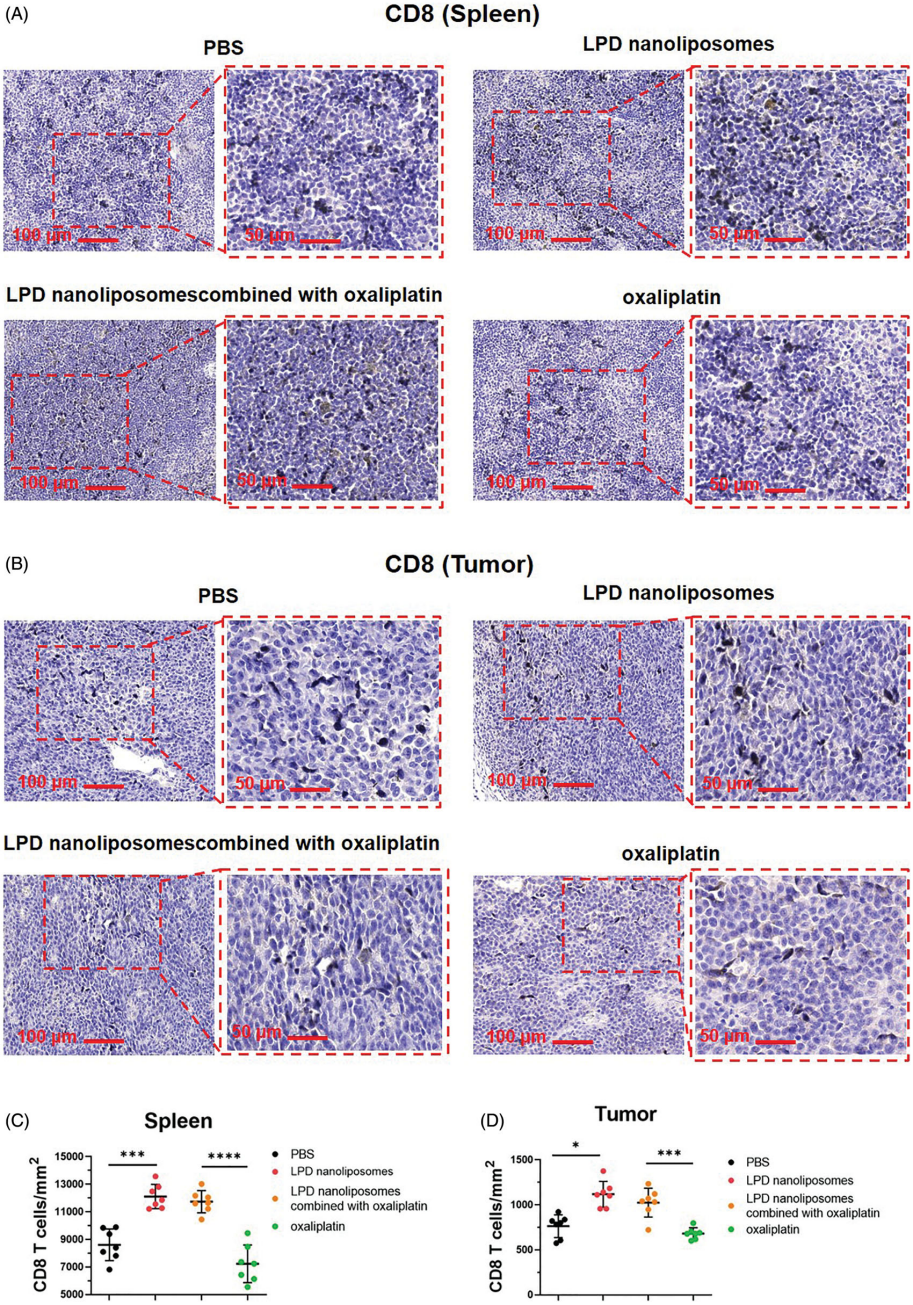
CD8+ T cell infiltration in tumors and spleen. (A) Representative images and quantitation of IHC staining of spleen for CD8. (B) Representative images and quantitation of IHC staining of tumor for CD8. (C) The number of CD8+ T cells mm^2^ of spleen tissue is shown. (D) The number of CD8+ T cells mm^2^ of tumor tissue is shown. The two groups are compared with two-tailed *t*-test. *p<.05; **p<.01; ***p<.001; ****p<.0001. Data are presented as means ± SD (*n* = 7).

CD8 + T cell infiltration can directly reflect the immune status of the body (Zhang et al., 2019; Sales de Sa et al., 2020; Cheung et al., 2021). In order to further explore whether LPD nanoliposomes can specifically enhance the body’s immune response to tumor cells and improve the body’s immune level, this study used tissue immunohistochemistry to detect each the infiltration of CD8 + T lymphocytes in spleen tissue and tumor tissue in the group. The results showed that the infiltration of CD8+ T cells in the mice spleen tissue from the LPD nanoliposome combined with oxaliplatin treatment group was higher than that of the oxaliplatin group (*p* < .0001 for both), and the infiltration of CD8+ T cells in the spleen tissue of mice in the LPD nanoliposome group was higher than that of the PBS group (*p* < .001 for both (Figure 7(A,C)) . In addition, the level of CD8 + T cell infiltration in tumor tissues was also measured. The results showed that the infiltration of CD8 + T cells in the tumor tissue of mice in the LPD nanoliposome combined with oxaliplatin group was higher than that in the oxaliplatin group (*p* < .001 for both), and the infiltration of CD8 + T cells in the tumor tissue of mice the LPD nanoliposome combined with oxaliplatin group was higher than that of the PBS group (*p* < .05 for both) (Figure 7(B,D)) . In short, the results indicate that mice immunized with LPD nanoliposomes showed stronger anti-tumor immunity.

## Discussion

Tumor vaccines have widely been a promising approach for cancer immunotherapy. However, many tumor patients are insensitive to these vaccines due to their weak immunogenicity as well as immunosuppression and identifying tumor-specific antigens can require expensive and laborious procedures. In this study, total tumor-derived RNA was extracted from CT-26 colorectal cancer cells and optimized encapsulated to construct LPD nanoliposomes. The results showed that LPD nanoliposomes showed tumor growth inhibition by activating the anti-tumor immune response in CT-26 colorectal cancer bearing mice, with minimal side effects toward the normal organs of mice. Furthermore, the effect of LPD nanoliposomes in combination with oxaliplatin can be better than that of oxaliplatin alone. Taken together, LPD nanoliposome vaccine loaded with tumor total RNA may serve as an effective antigen specific vaccine to induce antitumor immunity, presenting a new treatment option for colorectal cancer.

Cationic liposomes are one of the most studied nanocarriers in gene therapy (Charbe et al., 2020). Our previous studies have demonstrated that LPD nanoliposomes can deliver siRNA efficiently and the high efficacy of LPD nanoliposomes was attributed to the high PEG density and sheddable PEG of LPD nanoliposomes (Gao et al., 2011, 2012, 2013). However, no studies have been carried out to evaluate the stability and transfection efficacy of LPD nanoliposomes delivering RNA. Our study shows that by optimizing the preparation method of LPD nanoliposomes, we have successfully prepared more superior LPD nanoliposomes with the average size of 120.37 ± 2.949 nm and zeta potential was 3.34 ± 0.056 mV, and more RNA loading. TEM images also demonstrated that the improved LPD nanoliposomes were evenly distributed, with spheric shape and a size of approximately 100 nm. Significantly, gel electrophoresis demonstrated that LPD nanoliposomes could protect the RNA well with good stability in the whole process of 5 days. The versatility of the improved LPD nanoliposomes in the efficient delivery of small-molecule siRNA and large RNA has greatly broadened the application of LPD nanoliposomes in gene therapy.

The evaluation of RNA transfection efficacy is important for the study of RNA delivery system. However, most studies mainly focus on the label of RNA with isotype or fluorescent molecule (Addison et al., 2010). However, these methods are complex, expensive and laborious. Furthermore, only the transfection efficacy results were obtained from isotype or fluorescent molecule labeled RNA, and the RNA expression level could not be obtained from these results (Paulines & Limbach, 2017). Notably, we constructed CT-26 cells stably expressing green fluorescent protein and extracted RNA from these stably transfected cells. Next, we have transfected the RNA (ZSGreen) loaded LPD nanoliposomes into CT-26 cells and evaluated their transfection efficacy. The results showed that LPD nanoliposomes could not only efficiently deliver RNA in CT-26 cells, but also the expression of RNA (reflected by the green fluorescence) was observed, suggesting that our prepared LPD nanoliposomes are highly efficient RNA delivery systems. As the content of ZSGreen was very less in total RNA, the green fluorescence intensity expressed by RNA (ZSGreen) LPD was weak after transfection of CT-26 cells, and the transfection efficiency was difficult to be determined by flow and other methods.

Subsequently, we evaluated the anti-tumor immune mechanism and tumor-inhibitory effect of LPD nanoliposome. We first used RNA (ZSGreen) LPD to transfect DCs, but unlike CT-26 cells, no green fluorescence expression was found in DCs after transfection. but we found through *in vitro* simulation that after RNA LPD stimulated DCs *in vitro* (Zhang et al., 2019), it was detected that the dendritic function promoted T cell activation and proliferation. Changes in proliferating cells (CD11) (Figure 4(C)), changes in CD8 + T cells in the spleen and tumor infiltration (Figure 7). As expected, LPD nanoliposomes could activate DCs and T cells, resulting in high cytotoxic effect against CT-26 cells. It is well known that the anti-tumor effect of tumor vaccines depends on their effective presentation of tumor antigens to sensitize and induce specific CTLs (Shahnazari et al., 2020). Hence, tumor vaccines based on predetermined tumor antigen face the inherent risk of inefficient antigen presentation, resulting in immunosuppression and potential immune evasion. Further, it requires laborious procedures to identify tumor-specific antigens. Thus, tumor vaccines based on antigens derived from whole tumor cells have been developed to overcome these limitations (Fan et al., 2017; Yang et al., 2018). As confirmed by the results of many clinical trials, this type of vaccine has shown broad prospects in cancer treatment (Fan et al., 2017; Yang et al., 2018). Thus, we isolated total RNA from CT-26 cells to promote the potency and durability of anti-tumor immunity. The results showed that LPD nanoliposomes could slow tumor growth inhibition by activating the anti-tumor immune response in CT-26 colorectal cancer bearing mice, with minimal side effects toward the normal organs of mice. Furthermore, the effect of LPD nanoliposomes in combination with oxaliplatin can be better than that of oxaliplatin alone. Considering that a simple tumor immunotherapy shows significant but limited therapeutic efficacy, oxaliplatin was combined with LPD nanoliposomes, since the combination of immunotherapy and chemotherapy with different treatment regimens has become a new trend in the treatment of cancer (Ito et al., 2020; Fu et al., 2020; Golchin et al., 2019).Our prepared the improved LPD nanoliposomes have great potential in the translation into clinical use. At present, though RNA-pulsed DC vaccines have shown promise in clinical trials, they are limited by short shelf-life, costly and tedious preparation process (Saxena & Bhardwaj, 2018). Alternatively, the production of LPD nanoliposomes is within <6 hours, thereby shortening the time required to generate a personalized vaccine. Moreover, all the main reagents (such as DOTAP) used to prepare LPD nanoliposomes have been used in several clinical trials to enhance drug delivery, with favorable safety profiles and can be engineered to upregulate innate and adaptive host immunity (Wood et al., 2020). Therefore, the improved LPD nanoliposomes have promising application prospects in the clinical application of tumor immunotherapy.

**Figure 8.**
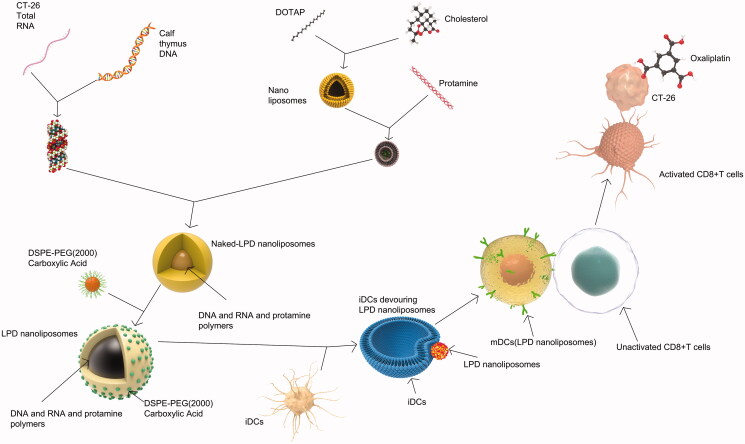
The preparation procedure and antitumor mechanism of LPD nanoliposomes. Nanoliposomes composed of DOTAP and cholesterol mixed with protamine and RNA and calf thymus DNA to form naked LPD nanoliposomes. After that, naked LPD was inserted with DSPE-PEG (2000) carboxylic acid to form LPD nanoliposomes. Immature DCs (iDCs) tends to swallow LPD nanoliposomes and become mature DCs (mDCs). These mDCs could activate CD8+ T cells and these activated CD8+ T cells are anticipated to specifically recognize and kill CT-26 tumor cells, combined with oxaliplatin.

Collectively, our data elucidated the antitumor mechanisms of LPD nanoliposomes in Figure 8. LPD nanoliposomes were prepared with DOTAP and cholesterol nanoliposomes, protamine, RNA and calf thymus DNA. Since iDCs are more likely to take up granular antigens, LPD nanoliposomes are expected to be taken up by iDCs, activating maturation of iDCs into mDCs. In the process of maturation process of DCs, it is anticipated that they will lose the ability to take up antigens but their antigen presentation ability to T cells gradually increases. When they are fully mature, they are expected to stimulate and activate T cells. After then, activated CD8 + T cells specifically recognize CT-26 cells and kill them. Oxaliplatin could greatly assist the cytotoxic effect of LPD nanoliposomes.

## Conclusion

RNA LPD nanoliposomes vaccines represent a promising treatment against cancer. In this study, the improved LPD nanoliposomes showed a high stability, with a low toxicity and high cell transfection efficacy toward CT-26 colorectal cancer cells. Notably, the improved LPD nanoliposomes showed tumor growth inhibition by activating the anti-tumor immune response in CT-26 colorectal cancer bearing mice, with minimal side effects toward the normal organs of mice. Thus, the improved LPD nanoliposome vaccine loaded with tumor total RNA may serve as an effective antigen specific vaccine to induce antitumor immunity, presenting a new treatment option for colorectal cancer.
